# Zfhx3-mediated genetic ablation of the SCN abolishes light entrainable circadian activity while sparing food anticipatory activity

**DOI:** 10.1016/j.isci.2021.103142

**Published:** 2021-09-16

**Authors:** Ashleigh G. Wilcox, R. Sonia Bains, Debbie Williams, Elizabeth Joynson, Lucie Vizor, Peter L. Oliver, Elizabeth S. Maywood, Michael H. Hastings, Gareth Banks, Patrick M. Nolan

**Affiliations:** 1 MRC Harwell Institute, Harwell Science Campus, Oxfordshire OX11 0RD, UK; 2 MRC Laboratory of Molecular Biology, Cambridge Biomedical Campus, Cambridge CB2 0QH, UK

**Keywords:** Biological sciences, Physiology, Molecular biology, Neuroscience

## Abstract

Circadian rhythms persist in almost all organisms and are crucial for maintaining appropriate timing in physiology and behaviour. Here, we describe a mouse mutant where the central mammalian pacemaker, the suprachiasmatic nucleus (SCN), has been genetically ablated by conditional deletion of the transcription factor *Zfhx3* in the developing hypothalamus. Mutants were arrhythmic over the light-dark cycle and in constant darkness. Moreover, rhythms of metabolic parameters were ablated *in vivo* although molecular oscillations in the liver maintained some rhythmicity. Despite disruptions to SCN cell identity and circuitry, mutants could still anticipate food availability, yet other zeitgebers - including social cues from cage-mates - were ineffective in restoring rhythmicity although activity levels in mutants were altered. This work highlights a critical role for *Zfhx3* in the development of a functional SCN, while its genetic ablation further defines the contribution of SCN circuitry in orchestrating physiological and behavioral responses to environmental signals.

## Highlights

• Deletion of Zfhx3 in developing hypothalamus leads to behavioral arrhythmicity
• SCN cell identity is absent while other retinal targets and visual functions remain
• Rhythms in metabolic functions are lost while some molecular rhythms in liver persist
• Conditional mutants can respond to food availability and other environmental cues

## Graphical abstract

**Figure:**
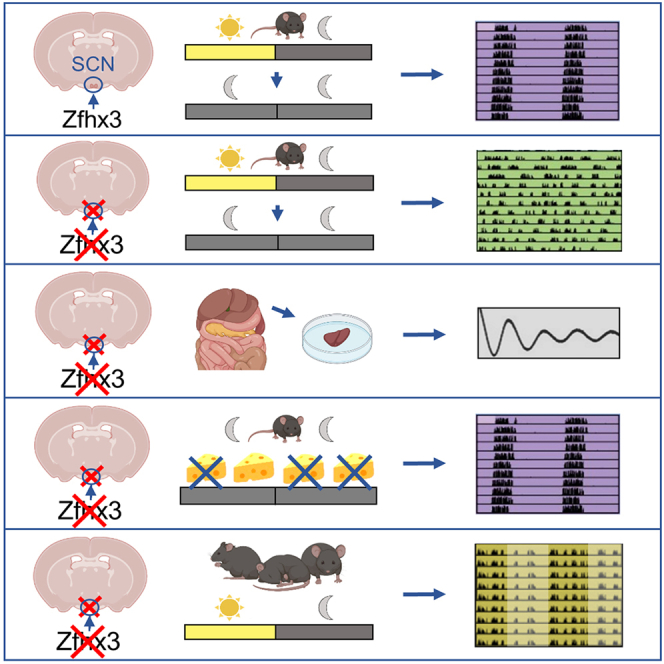

## Introduction

Circadian rhythms are endogenous 24-hour oscillations present in almost all organisms (Dunlap, 1999). In mammals, cell autonomous rhythms are formed by canonical transcriptional-translational feedback loops incorporating numerous well-characterized clock genes. Subsequently, cellular clocks at tissue and organ levels are maintained and synchronized by a central circadian oscillator in the hypothalamus: the suprachiasmatic nucleus (SCN). Peripheral tissue oscillators, additionally, relay cues back to the SCN and, in doing so, form robust circadian clocks both at the level of individual tissues/organs and the organism (Weaver, 1998). Furthermore, entrainment of an organism's internal circadian rhythms to the external environment is a critical feature. For animals, the strongest entraining factor (or *Zeitgeber*) is light, whereas other factors, such as food availability and social cues, can also play significant roles (Refinetti, 2015). Maintaining and resetting this synchronous activity is critical for health and well-being. Desynchrony has been shown to have adverse physiological consequences both in animal models and humans (Jagannath et al., 2017; Roenneberg and Merrow, 2016) and is a known hallmark of many psychiatric disorders (Banks et al., 2016; Ben-Hamo et al., 2016; Landgraf et al., 2016).

The canonical molecular elements of cell-autonomous clocks are well established. In mammals, the protein products of period (*Per*) and cryptochrome (*Cry*) genes negatively regulate their own transcription by inhibiting the action of positive transcriptional regulators CLOCK and BMAL1 (Takahashi, 2017). A second feedback loop includes the regulatory element Rev*-*erbα, whose protein product can repress *Bmal1* transcription. In addition to these canonical elements; however, a number of transcription factors contribute to the regulation of the circadian clock in SCN and other organs. Factors such as RORA (Sato et al. 2004), DBP (Yamaguchi et al., 2000), DEC1 (Nakashima et al., 2008) and DEC2 (Honma et al., 2002) have long-established roles in clock function. Moreover, transcription factors that are critical for SCN development and maintenance of SCN cell identity have recently also been shown to have important clock functions. For example, the Lim-domain homeobox gene *Lhx1* is critical for terminal differentiation of SCN neurons in mice. Deletion of this gene in the developing SCN results in moderate clock disturbances, including damping of SCN molecular rhythms and highly variable rhythms of wheel-running activity in constant darkness (Bedont et al., 2014). Whether these effects are a general characteristic of disturbed SCN development or reflect specific consequences of disturbed transcription factor function remains to be determined.

The gene *Zfhx3* is highly expressed in the developing brain before undergoing rapid downregulation in the majority of regions following birth (Ishii et al., 2003). In mice, *Zfhx3* is expressed in the SCN from as early as E13.5 (VanDunk et al., 2011). In the adult, expression is maintained in a small number of discrete brain nuclei, including the SCN, where gene expression remains strikingly prevalent. Previous work on this gene has highlighted the importance of ZFHX3 in the control of adult circadian rhythms. Although homozygous embryonic lethal, a dominant missense mutation in *Zfhx3* (short circuit, *Zfhx3*^*Sci/+*^) causes heterozygous mice to express a short circadian period when free-running in constant darkness and gives rise to altered expression of neuropeptide neurotransmitters and receptors critical for intercellular signaling within the SCN (Parsons et al., 2015). These effects are not purely dependent on developmental expression of *Zfhx3*, however, because a sustained role for *Zfhx3* in adult SCN has been established using a specific inducible deletion of *Zfhx3* where loss of the gene in adult mice acutely shortens wheel-running period or causes arrhythmicity (Wilcox et al., 2017).

As constitutive null *Zfhx3* mutations result in lethality, here we generated conditional null mice with spatially restricted deletion in the developing anterior hypothalamus to investigate its role in the SCN from its earliest stage of expression. Mutant animals lacking *Zfhx3* in this region were viable and fertile, yet surprisingly they lacked SCN structural morphology or cell identity. In locomotor behavioral assays these mutants were arrhythmic under both constant conditions and in a light-dark (LD) cycle despite normal visual acuity and retinal function. Interestingly, mutants were able to entrain to a scheduled feeding time, indicating that the food entrainable oscillator (FEO) (Pendergast and Yamazaki, 2018) was intact and does not require a functional SCN. Furthermore, when mutants were housed with wild-type littermates, the activity of a proportion of animals demonstrated low amplitude rhythms and showed a correlation with the activity patterns of their littermates. In summary, we demonstrate an essential role for ZFHX3 in establishing SCN cell identity. The intersectional mouse mutant generated in these studies proves to be a unique and robust model and will be an invaluable asset to future studies in circadian neurobiology. In contrast to SCN lesioning studies, this mutant enables a precise and complete elimination of SCN neuronal function from its earliest stages while surrounding cellular function and circuitry are apparently unaffected.

## Results

### Conditional deletion of Zfhx3 in SCN results in its genetic ablation

**Figure 1:**
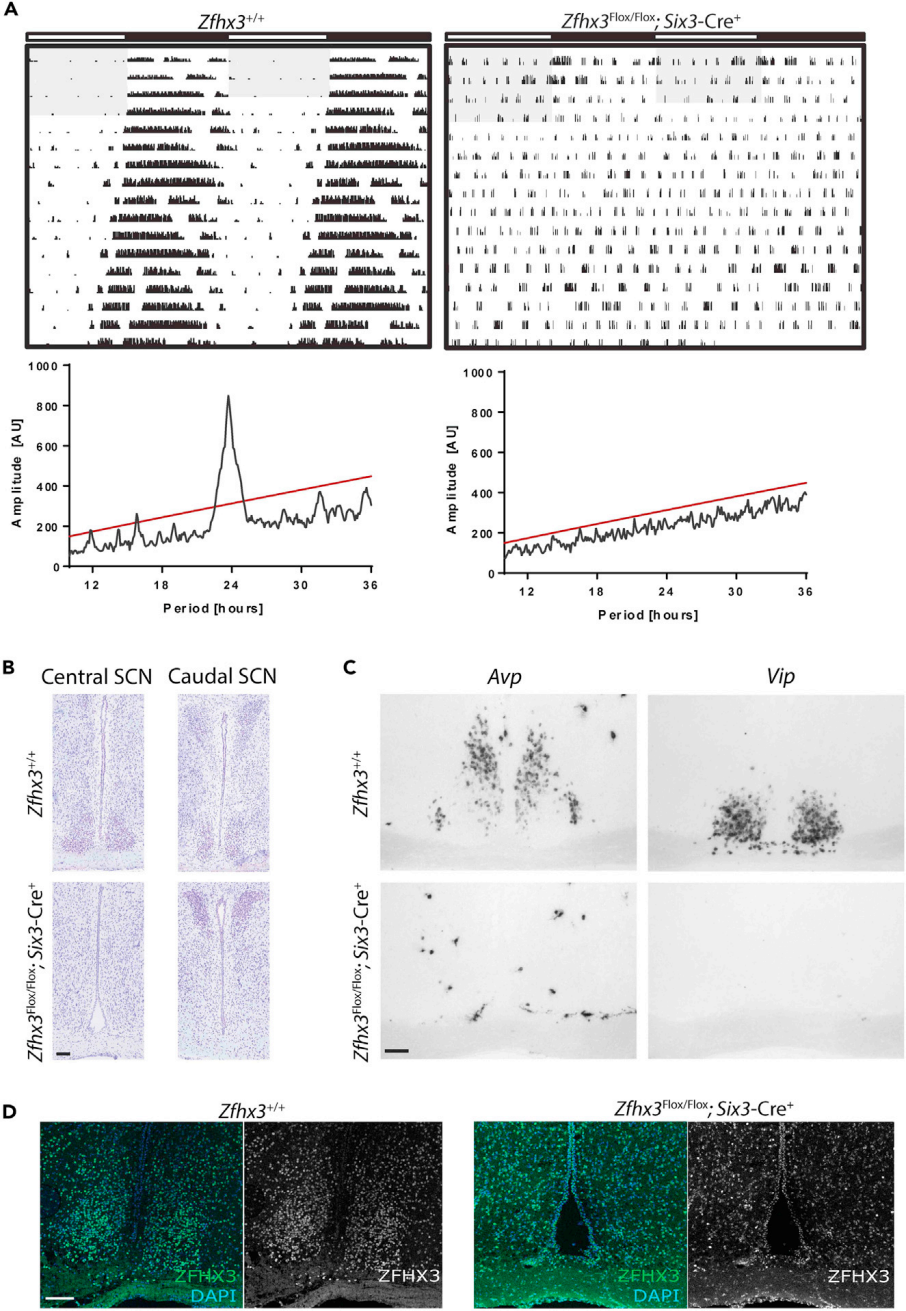
Arrhythmia and SCN anomalies in *Zfhx3*^Flox/Flox^; *Six3*-Cre^+^ mutant mice (A) Representative double-plotted actograms for *Zfhx3*^Flox/Flox^; *Six3*-Cre^-^ control (left) and *Zfhx3*^Flox/Flox^; *Six3*-Cre^+^ mutant animals (right) (A). Animals were initially housed in 12:12 light-dark (LD) cycles for five days and then subjected to two weeks of constant darkness (DD). Shaded parts of actograms represent lights-on, wheel-running is represented as vertical black bars. Periodograms of these representative plots, generated for the DD portion of the recording, are indicated underneath each actogram. (B) Scale bar 100 μm, Nissl-stained sections through the anterior hypothalamus showing the lack of cell clustering in the region of the SCN of *Zfhx3*^Flox/Flox^; *Six3*-Cre^+^ mutant brain while this is evident in control slices (B). (C) Scale bar 100 μm, Note that the PVN is intact in mutants. Representative *in situ* hybridizations (n = 3) of *Vip* and *Avp* in sections of *Zfhx3*^*+/+*^*; Six3-Cre*^*+*^and *Zfhx3*^Flox/Flox^; *Six3-Cre*^*+*^brains (C). (D) Scale bar 100 μm, Representative immunofluorescence staining for ZFHX3 in *Zfhx3*^+/+^ SCN while this is not evident in *Zfhx3*^Flox/Flox^; *Six3*-Cre^+^ sections (D). Scale bar 100 μm. See also Figures S1–S3.

To generate mice deleted for *Zfhx3* in the SCN, we crossed *Zfhx3*^Flox/+^ mice to a transgenic line expressing *Cre* driven by the *Six3* promoter. This line expresses *Cre* in the ventral hypothalamus (Furuta et al., 2000), overlapping with strong *Zfhx3* expression in the SCN (Parsons et al., 2015). Homozygous deleted *Zfhx3*^Flox/Flox^; *Six3*-Cre^+^ animals and littermate controls were subjected to behavioral, molecular and cellular analysis. Screening for differences in circadian wheel-running parameters revealed a severe and reproducible circadian phenotype: arrhythmic locomotor behavior in both LD and constant dark (DD) conditions. Mice displayed no rhythmic activity in DD, indicating the lack of a functional internal clock. This was accompanied by a complete absence of circadian responsiveness to light evident in the lack of negative masking and arrhythmicity in LD conditions (Figures 1A, S1A). This phenotype was present in both male and female animals from as early as six weeks of age. To confirm whether the activity of *Zfhx3*^Flox/Flox^; *Six3*-Cre^+^ animals was truly arrhythmic, Cosinor analysis was used to establish whether rhythms were detectable in the activity cycles of these animals. We found no significant rhythms in any of the mutant animals analysed. Notably, when the light:dark activity of *Zfhx3*^Flox/Flox^; *Six3*-Cre^+^ animals was compared to that of *Zfhx3*^Flox/Flox^; *Six3*-Cre^-^ animals, pairwise comparisons of activity in 30 min time bins revealed that all significant differences in activity were present exclusively in the dark phase of the cycle (repeated measures ANOVA, interaction factors “Wheel running counts x ZT”; p < 0.05 for ZTs 12 to 20). We found no significant differences in activity in the light phase (Figure S1B). Thus, a failure to initiate sustained activity in the dark phase/subjective night was a defining feature of homozygotes. To investigate whether this phenotype was due to a change in the circadian response to light, we also conducted analysis of the animal’s ability to mask in response to a light pulse in the dark phase. Because of the reduced wheel running activity of *Zfhx3*^Flox/Flox^; *Six3*-Cre^+^ animals, it was difficult to investigate using wheel running screens. Since running wheel analysis only quantifies voluntary activity, we therefore used video tracking to more accurately measure activity during a masking light pulse. Here animals were given a one hour light pulse at ZT14 and their movement monitored prior to and following the light pulse (Figure S2). Statistical analysis demonstrated that while control animals significantly reduced their activity during the light pulse (one way ANOVA, F(20,126) = 3.66, p = 0.0001), *Zfhx3*^Flox/Flox^; *Six3*-Cre^+^ animals showed no significant change in their activity throughout the period of the light pulse (one way ANOVA, F(20,105) = 1.4, p = 0.1402). *Zfhx3*^Flox/+^; *Six3*-Cre^+^ animals (i.e., heterozygous null animals) were also tested in circadian screens and found to be no different from rhythmic, wild-type littermate controls (Figure S3). Subsequent histological examination revealed a characteristic ‘teardrop’ SCN morphology in control animals lateral to the third ventricle and above the optic chiasm. In contrast, there was no evidence of any such structure in the suprachiasmatic hypothalamus of homozygous mutants (Figure 1B). Furthermore, Nissl-stained hypothalamic morphology both dorsal to the SCN (including paraventricular nucleus) and caudal to the SCN (including arcuate nucleus) remained intact in mutants. A lack of terminally differentiated SCN cell identity was confirmed using *in situ* hybridization and immunofluorescence. Homozygous animals showed no expression of transcripts for key SCN neuropeptide genes *Vip* and *Avp,* typically used to delineate the ventrolateral core and dorsomedial shell of SCN (Yan et al., 2007) (Figure 1C)*.* As expected, immunostaining for ZFHX3, normally highly expressed in the SCN, showed no characteristic dense immunoreactivity in mutants, while the ZFHX3 signal in surrounding regions showed the expected diffuse nuclear staining similar to that seen in wild-type animals (Figure 1D). These observations reveal a developmentally specific role for ZFHX3, and also show that a competent SCN is necessary to sustain circadian behavioral rhythms by its promotion of activity, rather than suppression of quiescence.

### Retinal innervation of SCN is absent in mutants while other retinal functions remain intact

**Figure 2:**
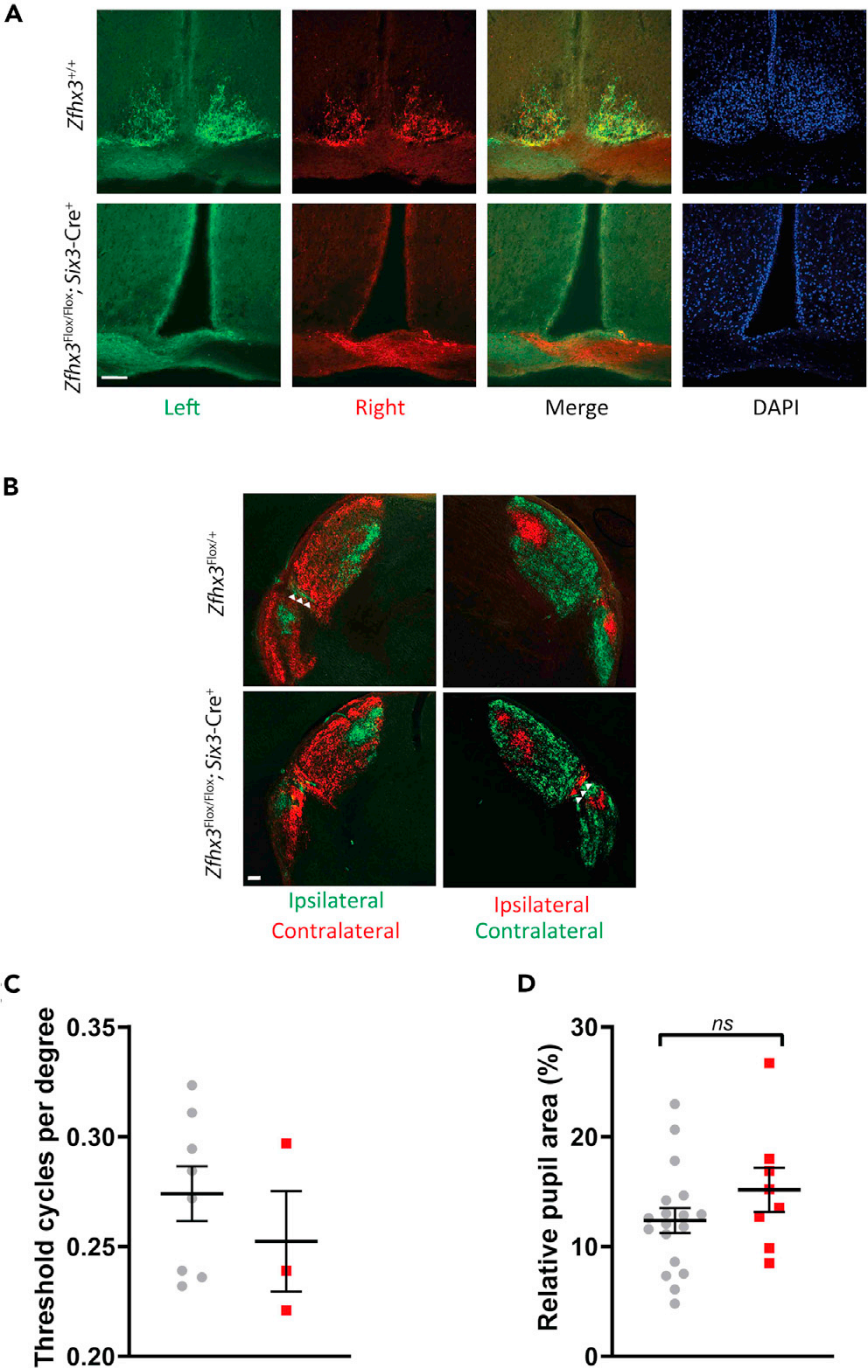
Visual functions spared in SCN-deficient *Zfhx3*^Flox/Flox^; *Six3*-Cre^+^ mutant mice Anterograde RGC projections following intravitreal injections of cholera toxin B subunit conjugated to Alexa Fluor 488 (left eye) and 594 (right eye) into the eyes of *Zfhx3*^*Flox/Flox*^*; Six3-*Cre^*+*^ and control mice reveal a complete lack of RGC axonal innervation to the suprachiasmatic region. (A) Scale bar 100 μm, Representative images of ipsilateral and contralateral projections to the SCN of a control mouse (top), which are completely lacking in the *Zfhx3*^*Flox/Flox*^*; Six3-Cre*^*+*^ mice (bottom) which have no anatomically distinct SCN (see DAPI image). (B) Scale bar 100 μm. However, both mice show the ipsilateral and contralateral projections from the RGCs to the LGN including IGL (arrowheads) (B). (C) Visual acuity was measured using an optokinetic drum. No differences in response were found in *Zfhx3*^*Flox/Flox*^*; Six3-*Cre^*+*^ (red, n = 3) and control mice (grey, n = 8) (C). (D) Percentage pupil area at the point of maximal constriction (relative to dark-adapted pupil size) following bright (100 lux) light stimuli to the eyes of control (gray, n = 18) and *Zfhx3*^*Flox/Flox*^*; Six3-Cre*^*+*^ (red, n = 8) mice (D). Mean ± SEM including individual data points. No significant differences were found between groups when analysed using ANOVA.

Given the apparent absence of behavioral masking by light, we sought to determine whether retinal function was also compromised in mutants and first looked at retinal ganglion cell (RGC) innervation of brain targets in both image-forming (lateral geniculate nucleus, LGN) and non-image-forming visual function centers (SCN) using anterograde tracing with cholera toxin B-488 and 594. The control mice showed the typical ipsilateral and contralateral retinofugal innervation of the SCN (Figure 2A top). However, there was no anatomically distinct SCN in the *Zfhx3*^Flox/Flox^; *Six3*-Cre^+^ mice (see Figure 2A DAPI bottom) and a complete lack of RGC axonal innervation to the suprachiasmatic region where the SCN would typically reside (Figure 2A bottom). Ipsilateral and contralateral innervation to the image-forming LGN region including the intergeniculate leaflet (IGL) was similar in the control and the *Zfhx3*^Flox/Flox^; *Six3*-Cre^+^ mice (Figure 2B). This suggested that the inability of the *Zfhx3*^Flox/Flox^; *Six3*-Cre^+^ mouse to entrain to an LD signal was due solely to loss of the SCN and a lack of establishment of SCN circuitry. It also suggested that retinal input to the LGN was not sufficient to mediate masking. We therefore explored whether other retinal functions were affected in mutants. Using an optokinetic drum, we established that visual tracking in mutants was still present (Figure 2C) while the consensual pupillary light response was comparable to controls (F(1,24) = 1.72; *p* = 0.3279) (Figure 2D). This provided further evidence that deficits in mutants were consequences of SCN loss alone and that retinal function and circuitry, excluding retinal-SCN afferents, were intact in *Zfhx3*^Flox/Flox^; *Six3*-Cre^+^ mice.

### Rhythms in metabolic output are absent in mutants while hepatic molecular oscillations are present but reduced

**Figure 3:**
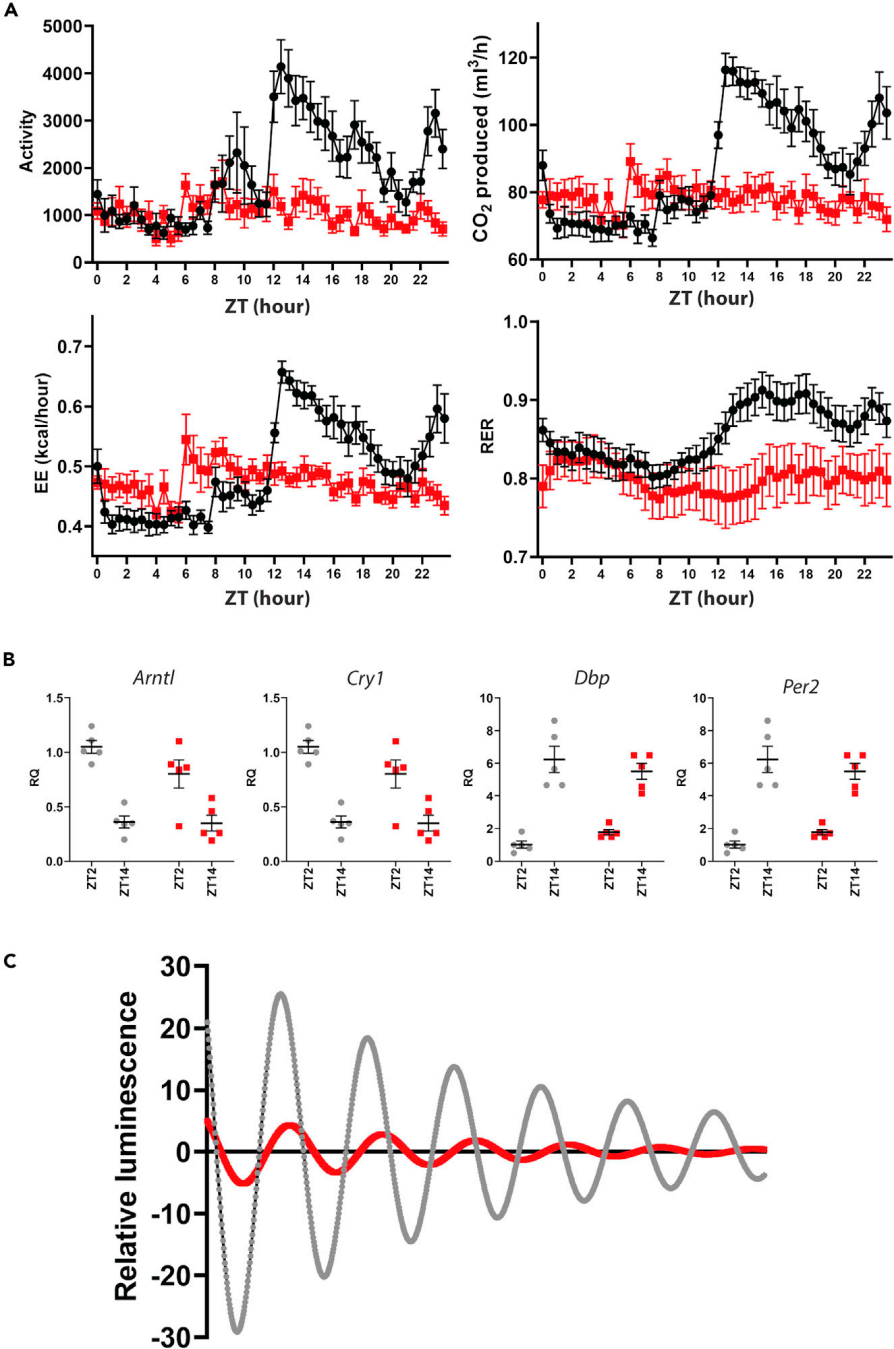
Metabolic and liver function in *Zfhx3*^Flox/Flox^; *Six3*-Cre^+^ mutant mice (A) Plots of mean (±SEM) hourly measures for activity, CO_2_ consumption, EE and RER in *Zfhx3*^Flox/Flox^; *Six3*-Cre^+^ [red, n = 8] and *Zfhx3*^Flox/Flox^ [black, n = 11] cohorts (A). (B) Levels of clock gene transcripts in control (n = 5, grey) and mutant livers (n = 5, red) collected at ZT2 and ZT14 (B). Mean (±SEM) and individual values are indicated. (C) Average traces showing Per2: Luc oscillations in liver slices from control (grey trace) and *Zfhx3*^Flox/Flox^; *Six3*-Cre^+^ (red trace) mutant mice (C). See also Figure S4–S6.

No body weight differences were evident in *Zfhx3*^Flox/Flox^; *Six3*-Cre^+^ mice. Furthermore, we found no significant differences in fat or lean mass in mutant males (Figure S4) and so overall metabolic regulation was unaffected by the deletion. We next investigated whether metabolic rhythms were evident in whole animals by housing them individually in metabolic cages. Visual inspection of data over 24 hours in 30 min bins suggested that *Zfhx3*^Flox/Flox^; *Six3*-Cre^+^ males lacked rhythms in activity, O_2_ consumption, CO_2_ expenditure, RER and EE (Figures 3A, S4, and S5). In comparison *Zfhx3*^Flox/Flox^; *Six3*-Cre^-^ control males exhibited clear rhythmicity with peaks at the transitions of lights-on and -off. Repeated measures ANOVA performed upon these metabolic data demonstrated that the majority of significant differences between genotypes were found in the dark phase of the light:dark cycle (repeated measures ANOVA, interaction factors “Metabolic parameter x ZT”; pairwise comparisons show significant (p < 0.05) differences in 70% of dark phase intervals and 10% of light phase intervals). To confirm that *Zfhx3*^Flox/Flox^; *Six3*-Cre^+^ mice were truly metabolically arrhythmic, Cosinor analysis was used to establish whether rhythms were detectable in the EE and activity data of *Zfhx3*^Flox/Flox^; *Six3*-Cre^+^ and *Zfhx3*^Flox/Flox^; *Six3*-Cre^-^ animals. For both EE and activity, none of the *Zfhx3*^Flox/Flox^; *Six3*-Cre^+^ showed significant rhythmicity (8/8 animals p > 0.05), whereas all of the *Zfhx3*^Flox/Flox^; *Six3*-Cre^-^ animals showed significant rhythms in both parameters (11/11 animals p < 0.05). Notably, despite this lack of metabolic rhythms, we detected no gross changes between genotypes in average daily O_2_ consumption (F(1,11) = 1.04; p = 0.32), average daily CO_2_ expenditure (F(1,11) = 0.78; p = 0.39), average daily energy expenditure (F(1,11) = 1.07; p = 0.32), body weight (F(1,12) = 0.11; p = 0.74), and fat mass (F(1,12) = 0.05; p = 0.82) or lean mass (F(1,12) = 0.02; p = 0.89) (Figure S4). To estimate any differences in local molecular oscillations, liver tissue was collected from *Zfhx3*^Flox/Flox^; *Six3*-Cre^+^ and control mice at two opposing circadian time points (ZT2 and ZT14) and expression levels of *Arntl*, *Cry1, Per2,* and *Dbp* evaluated using qRT-PCR. We found no significant differences between genotypes in the expression of these genes (2-way ANOVA, interaction factors “RQ x ZT”; p > 0.05; *Arntl* F(1,1) = 0.63; *Cry1* F(1,1) = 0.21; *Per2* F(1,1) = 4.6; *Dbp* (F1,1) = 2.41) suggesting that molecular rhythms in the liver of *Zfhx3*^Flox/Flox^; *Six3*-Cre^+^ animals were still present (Figure 3B). In addition, we compared *ex vivo* oscillations in liver slices from mutants and controls using the *Per2::Luc* reporter line. Although a comprehensive analysis of these data was difficult because of high variability between different trials, visual inspection of samples from within the same trial suggested that oscillations from *Zfhx3*^Flox/Flox^; *Six3*-Cre^+^ animals were present but severely damped compared to controls (Figures 3C and S6). A basic analysis of the data demonstrated that the magnitude of the first peak of the rhythms of *Zfhx3*^Flox/Flox^; *Six3*-Cre^+^ animals was reduced (luminescence signal of first peak: control = 38.9 ± 14; *Zfhx3*^Flox/Flox^; *Six3*-Cre^+^ = 6.8 ± 1; p = 0.046). Furthermore we found no significant change either in the rate of peak decay (loss of peak signal per cycle: control = 27.1 ± 4.1%; *Zfhx3*^Flox/Flox^; *Six3*-Cre^+^ = 44.1 ± 6.9; p = 0.11) or in the peak to peak period (average peak to peak time: control = 22.5 ± 0.5 hours; *Zfhx3*^Flox/Flox^; *Six3*-Cre^+^ = 23.5 ± 0.3 hours; p = 0.13).

### Anticipation of feeding times remains intact in mutants

**Figure 4:**
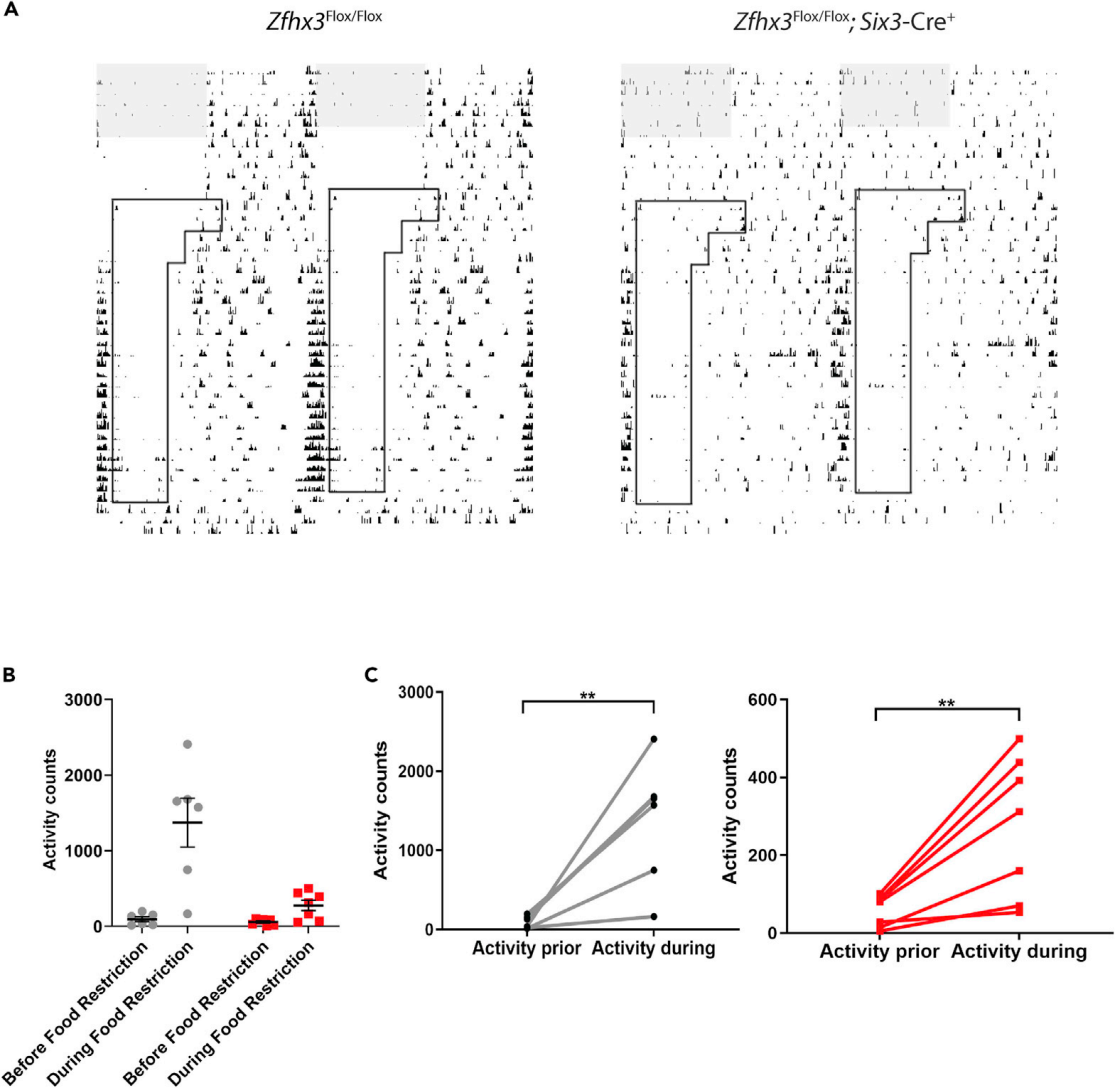
*Zfhx3*^Flox/Flox^; *Six3*-Cre^+^ mice anticipate food availability (A) Representative actograms for control (*Zfhx3*^Flox/Flox^; *Six3*-Cre^-^) and *Zfhx3*^Flox/Flox^; *Six3*-Cre^+^ mice undergoing a time-restricted feeding protocol (A). Mice were maintained on *ad libitum* food in a 12:12 LD cycle before being transferred to DD where food availability was restricted, shaded regions indicate lights-on, boxed regions denote periods of food availability during the food restriction protocol, short vertical bars indicate wheel-running. (B and C) Total activity counts for 3-hour time windows immediately preceding time of food availability in *Zfhx3*^Flox/Flox^; *Six3*-Cre^+^ (n = 7), and *Zfhx3*^Flox/Flox^ (n = 7) mice on days prior to (left) and during (right) time restricted feeding (B, C). Means and individual values shown. Error bars denote SEM of datasets; ∗∗ denotes significance at *p* < 0.01 when analysed using ANOVA.

To measure the output of the FEO, food anticipatory activity (FAA) was measured in *Zfhx3*^Flox/Flox^; *Six3*-Cre^+^ and control mice using a modified wheel-running paradigm. Following release into DD, food availability was restricted to a 6-hour window and we measured FAA activity in a three-hour interval immediately prior to food access. Under *ad libitum* feeding conditions, mutant animals were arrhythmic as expected. Under food restriction, however, robust FAA became evident in *Zfhx3*^Flox/Flox^; *Six3*-Cre^+^ animals, as it did in controls (Figure 4A). Although total activity levels were certainly lower in mutants compared to controls (adjusted p *=* 0.0072) under the restricted feeding regime (Figure 4B), the increase in activity counts measured in the FAA window during food restriction was significant (t(6) = 4.01, p = 0.007) and was comparable to that observed in controls (Figure 4C).

### Entrainment to social cues is not evident in mutants

**Figure 5:**
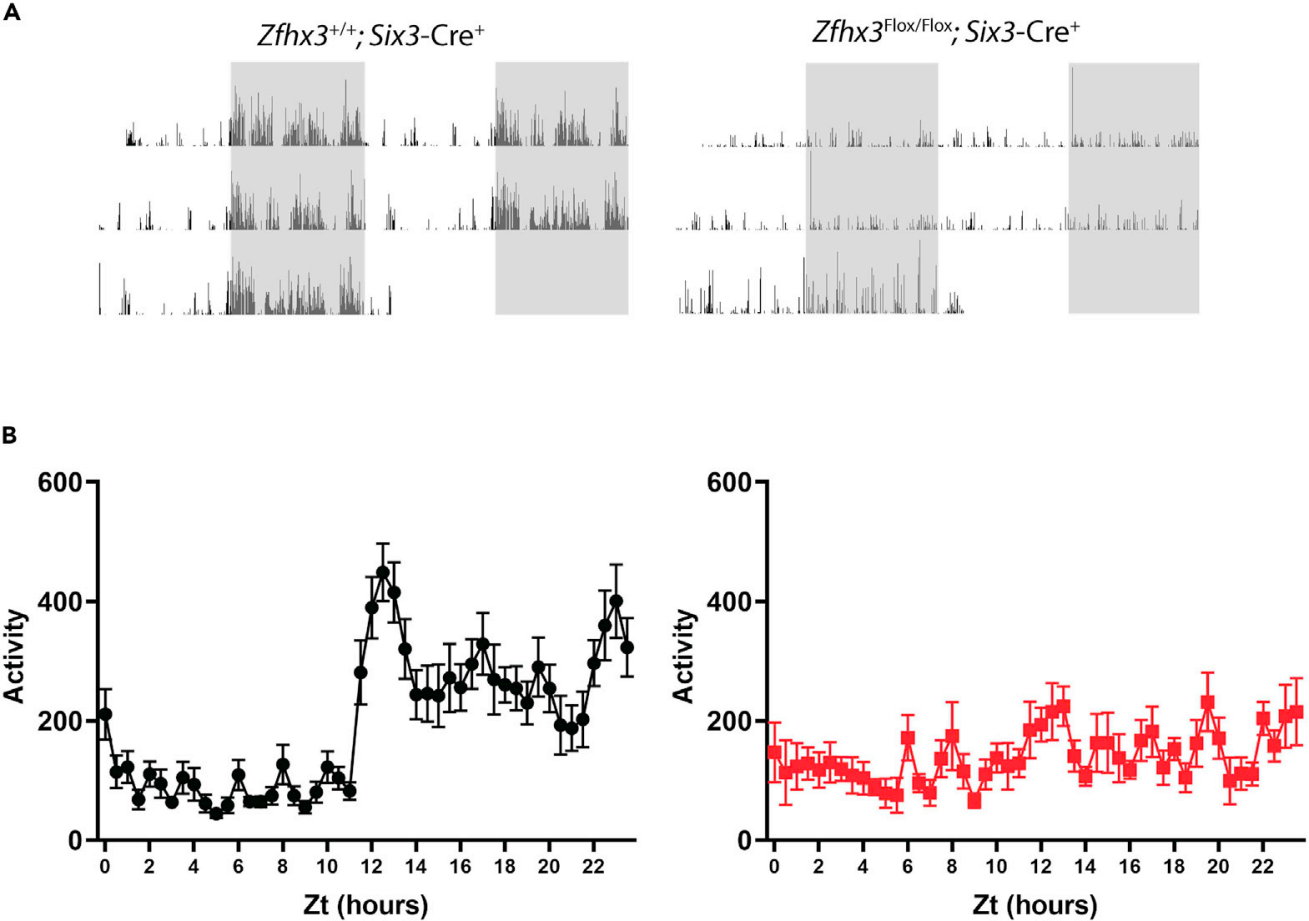
Influence of social housing in *Zfhx3*^Flox/Flox^; *Six3*-Cre^+^ mice (A) Representative actograms of control *Zfhx3*^+/+^; Six3-Cre^+^ (left) and *Zfhx3*^Flox/Flox^; *Six3*-Cre^+^ (right) animals co-housed under normal LD conditions (A). (B) Activity (distance travelled per time window) is represented as vertical black bars; shaded bar represents LD cycle. Line graphs showing average hourly measures for activity in *Zfhx3*^Flox/Flox^; *Six3*-Cre^+^ (red, n = 7), and *Zfhx3*^+/+^; Six3-Cre^+^ (black, n = 7) mice (B). Mean ± SEM for each time bin is shown. See also Figure S7.

Detailed studies of social entrainment in mice have been limited because of the practical difficulties of measuring individual activities in a group-housed environment over prolonged periods. To overcome this, we used the HCA recording system that makes use of implanted telemetric chips to record the activities of individual animals in group-housed conditions (Bains et al., 2016, 2018). *Zfhx3*^Flox/Flox^; *Six3*-Cre^+^ mice were weaned into cages with two control cage-mates and home-cage activity recorded over 3 days in LD conditions. Visual inspection of individual actograms revealed typical nocturnal activity in control animals with the expected increase in activity coinciding with the onset of the dark phase (Figure 5A). Conversely, mutant animals showed no increase in nocturnal activity and no clear patterns of rhythmicity as previously shown in the singly housed wheel-running assay. Statistical comparisons of the activity patterns of control and mutant animals (in 30 min time bins) confirmed that *Zfhx3*^Flox/Flox^; *Six3*-Cre^+^ animals lacked the nocturnal activity peak present in control animals (Figure 5B), as significant differences were found only in the dark phase of the light:dark cycle (repeated measures ANOVA, interaction factors “Activity x ZT”; pairwise comparisons show significant (p < 0.05) differences for intervals ZT12 to 14, ZT16 to 18 and ZT23). This lack of nocturnal activity is not a consequence of deficits in motor function as *Zfhx3*^Flox/Flox^; *Six3*-Cre^+^ animals showed normal locomotor activity measures in an open field test (Figure S7). In order to confirm the lack of rhythmic activity in mutant animals, the daily activity was analyzed using Cosinor analysis. Surprisingly, in this analysis 42.8% (3/7) of the mutants showed significant activity rhythms. To ascertain whether this apparent increase in rhythmicity in the *Zfhx3*^Flox/Flox^; *Six3*-Cre^+^ animals was because of the influence of their control cagemates, we performed a correlation analysis of the daily activity of each animal against its cage mates. In this analysis we found significant (p < 0.05) correlations between the activity patterns of all animals within the same cage, regardless of genotype. Notably the correlation coefficient of control vs *Zfhx3*^Flox/Flox^; *Six3*-Cre^+^ animals did not significantly differ from that of control vs control animals (average Pearson correlation coefficient: control vs control = 0.69 ± 0.044; control vs *Zfhx3*^Flox/Flox^; *Six3*-Cre^+^ = 0.601 ± 0.041; p = 0.143). Furthermore the coefficients denoted a positive correlation between the activity patterns of animals within the same cage regardless of genotype.

## Discussion

Transcription factors serve critical roles in establishing and maintaining cellular identity, circuitry and function in the central nervous system. Consequently, in order to dissect their roles in these processes, it is important to study the effects of multiple allelic mutations in each gene to establish their distinct temporal and region-specific roles in neuronal function. In previous work, we have established a role for the transcription factor ZFHX3 in the maintenance of SCN function using both a missense mutation and adult-specific conditional deletion of the gene (Balzani et al., 2016; Parsons et al., 2015; Wilcox et al., 2017). ZFHX3 is, however, highly expressed in the developing brain but because early deletion of this gene results in early postnatal lethality in heterozygotes, it has been difficult to separate its roles in the adult from its functions in establishing the circuitry of the developing nervous system. In this study we generated a spatially restricted deletion of *Zfhx3*, using a Six3-Cre mouse line that expresses Cre in the ventral anterior hypothalamus but lacks Cre activity in more posterior regions of the brain (Bedont et al., 2014). Deletion of *Zfhx3* using this Cre line results in complete behavioral arrhythmicity in all lighting conditions and, correspondingly, there is no evidence of any terminally differentiated SCN structure. This effect is far more severe than that observed when other transcription factors in this region are deleted (Bedont et al., 2014) and highlights the critical role of ZFHX3 in establishing SCN function and circuitry. This lack of functionality was highlighted by an absence of neurons in the region that were positive for either *Vip* or *Avp*. Moreover, tracer studies showed no functional afferents from the retina to the region of the hypothalamus including the SCN whereas other retinal afferents remained intact.

Deletion of *Zfhx3* by Six3-driven Cre activity within the hypothalamus is limited to the SCN while ZFHX3 expression remains comparable to control animals in hypothalamic regions including the paraventricular nucleus and arcuate nucleus. This afforded us an opportunity to investigate how lack of integrity of the SCN might influence biological rhythms in the periphery. In comparison to lesion studies, genetic ablation of the SCN enables a precise and comprehensive impairment of the region. Previous studies with SCN-lesioned mice would suggest that rhythms in metabolic function are damped (Coomans et al., 2013) but we wanted to confirm this using the more precise form of genetic ablation used here. Furthermore, SCN lesion studies are only established in adulthood after all appropriate circuitry has been established, whereas genetic ablation may not enable this circuitry to develop in the first instance. Initial results showed no differences in body weight, fat mass or lean mass in mutants and so metabolic homeostasis, per se, is not affected by SCN deletion. However, our data echo those from surgical SCN lesion studies, where parameters such as O_2_ consumption, CO_2_ production, energy expenditure and activity had no discernible rhythm. All rhythms appeared to be “locked” into measures that are consistent with the rest phase of the animal rather than showing intermediate measures. This finding is consistent with a recent study where all SCN neurons are inactivated using a Syt10-Cre driver line although partial limitation of SCN function either through inactivation of some of its neuronal constituents or through acute intervention of SCN neuronal function can result in increased activity (Houben et al., 2014; Collins et al., 2020). Thus, SCN-dependent metabolic control revolves around activation to sustain SCN-dependent behavioral arousal. Notwithstanding the loss of metabolic cycles in the mouse, our data show damped but significant oscillations in bioluminescent reporters in the livers of our mutant animals. In line with this we also show that the expression of various circadian genes at two opposing time points is unchanged in the livers of our transgenic animals. Taken together these data strongly suggest evidence of extra-SCN functionality. Notably, a recent study has highlighted the ability of hepatocytes *in vivo* to maintain both rhythmicity and phase coherence in the absence of an intact SCN or other oscillatory cells (Sinturel et al., 2021). The data reported here demonstrates that such hepatic rhythms persist (albeit damped) even following the developmental ablation of the SCN, suggesting that behavioral cycles may play an important amplifying role in the maintenance of local molecular rhythms. We do, however, also note that the rhythmicity observed in our bioluminescence studies could also be induced by dissection of the liver. Although it is unlikely that such dissection effects would induce the differences in gene expression we observe, further studies such as *in vivo* analysis of hepatic oscillations are required to clarify fully the presence and significance of circadian function in the liver of this developmental model.

Having established that the circadian activity of mutant animals fails to respond to environmental light input, we tested the ability of mutants to respond to other environmental signals where the role of the SCN is still debatable. The FEO has been extensively studied in mammals and still the exact location of this important oscillator remains elusive (Pendergast and Yamazaki, 2018). It is likely that the FEO consists not of one specific anatomical region, but a network of interconnected brain regions and peripheral oscillators. Furthermore, the relationship of the FEO oscillator with the SCN is debatable. Previous studies have suggested that the FEO is still functional in SCN ablated animals (Davidson, 2009) and in mutants of genes critical for SCN function (Fuller et al., 2008; Sheward et al., 2007). However, others have reported contrasting findings and methodological inconsistencies in such studies that suggest that the SCN does play an important role within the FEO (Mistlberger et al., 2008, 2009). Recent studies also point to important neuropeptide Y (NPY) afferents to the SCN from the intergeniculate leaflet and indicate that SCN integrity is still necessary for FEO function (Fernandez et al., 2020). The data we present here clearly demonstrates that animals lacking an SCN can show food anticipatory activity (FAA) and are therefore capable of integrating and responding to timing cues to some degree. Given that SCN surgical ablation inevitably compromises other local and possibly unrelated pathways that may affect FAA, the maintained FAA in the non-circadian, genetically SCN-deficient mouse argues against a role for the SCN and its dependent outputs in food timing cues. However, although the FAA shows the ability to anticipate timing cues, it is not itself evidence of entrainment and it is therefore unclear at present how the FEO is affected by the developmental loss of the SCN. Further, more detailed, investigations are required to establish the consequences to the FEO in this developmental model.

The *Zfhx3*^Flox/Flox^; *Six3*-Cre^+^ mutant line also gave us the opportunity to determine whether social zeitgebers are capable of entraining in the absence of the SCN. The idea of a social clock has been proposed in both rodents (Panksepp et al., 2008; Paul et al., 2014) and primates (Bessa et al., 2018), although the nature and strength of social zeitgebers has been disputed (Refinetti et al., 1992). In group-housed conditions, hamsters can adjust their circadian period in DD to that of their cage-mates (Paul and Schwartz, 2007) and these findings were used as a template for the current investigations. Using the HCA system, we were able to explore whether mutant animals could entrain to the social cues of wild-type cage-mates. We found that wild-type cage-mates showed strong, high-amplitude rhythms in a social setting, whereas mutants that had been weaned into the same cage lacked the nocturnal elevation of activity of their control cagemates. Despite this, rhythmicity analysis demonstrated that 42.8% of mutant animals displayed significant rhythmicity in their activity. Further analysis of these activity patterns suggested that mutant animals showed significant correlation with the activity patterns of their control animal cagemates. We note that, although this correlation suggests that animals within the same cage are active at similar times, this may be independent of the absolute level (or magnitude) of their activity. Further work is required to demonstrate whether this correlation represents social entrainment or whether it is simply the result of individual disturbances within the cage.

The data presented here describe the essential role of the transcription factor, ZFHX3, in laying down the cellular developmental blueprint for the mammalian circadian oscillator, the SCN. Moreover, the *Zfhx3*^Flox/Flox^; *Six3*-Cre^+^ mutant line offers an alternate approach to lesion studies whereby SCN function can be interrogated more precisely and consistently in the adult. This genetic ablation model provides us with the means to investigate the SCN as a self-sustaining oscillator whilst simultaneously investigating the roles and consequences of its numerous efferent and afferent circuitries.

### Limitations of the study

A limitation of this study is shared with any using a Cre driver line in a conditional deletion study. In many instances, Cre expression in cell lineages, developmental stages and, indeed, in specific adult cells and tissues could be overlooked or missed. This can often lead to data mis-interpretation. We and others (Furuta et al., 2000; Bedont et al., 2014) have mapped Cre expression in this driver line as fully as possible using Cre reporter lines but cannot categorically state that some expression in cells or lineages has been missed. Although we do not see any additional gross deficits in mutant animals, we cannot categorically state that minor additional deficits in mutants might be overlooked. Secondly, we do find molecular oscillations in mutant liver but, in retrospect, it would be useful to refine how these oscillations differ from wild-type. Finally, although we did find that mutants display food anticipatory activity, further work would be required to establish whether *Zfhx3* deficiency alters entrainment.

## STAR★Methods

### Key resources table

**Table:** 

REAGENT or RESOURCE	SOURCE	IDENTIFIER
**Antibodies**		

Rabbit ANti-ZFHX3, custom generated antibody	Parsons et al. (2015)	N/A
Goat Anti-Rabbit, Alexa 488 conjugated	Abcam (UK)	Cat#ab150077; RRID:AB_2630356
Anti-Digoxigenin-AP, Fab fragments from sheep	SigmaAldrich (Roche)	Cat# 11093274910; RRID:AB_514497

**Bacterial and virus strains**		

Cholera Toxin subunit B conjugated with Alexa Fluor 488	ThermoFisher Scientific	Cat#C34775
Cholera Toxin subunit B conjugated with Alexa Fluor 594	TheromFisher Scientific	Cat#C34777

**Critical commercial assays**		

Maxwell LEV SimplyRNA Tissue kit	Promega, UK	Cat#AS1280
High Capacity cDNA RT kit	ThermoFisher Scientific	Cat#4368814
Fast SYBR Green Mastermix	ThermoFisher Scientific	Cat#4385610

**Experimental models: organisms/strains**		

*Zfhx3*^*Tm1.1Jtd*^ Mouse line	Provided by Dr Jin-Tang Dong (Emory University) Sun et al., 2012	MGI:5446504
*Tg(Six3-cre)69Frty* Mouse line	Provided by Dr J Bedont (Johns Hopkins University), originally from Dr Yas Furuta (Furuta et al., 2000)	JAX stock Cat#019755
*Per2*^*Tm1Jt*^ Luciferase reporter line	Provided by Dr J Takahashi (Yoo et al., 2004)	JAX stock Cat#006852

**Oligonucleotides**		

PCR primers	See Table S1 for primers	N/A
Recombinant DNA		
pCR4-TOPO	ThermoFisher Scientific	Cat#450071

**Software and algorithms**		

Clocklab, data collection and analysis for circadian biology	Actimetrics (IL, USA)	N/A
AnyMaze	Stoelting Europe (Dublin, Ireland)	N/A
Ethovision XT	Noldus ( Wageningen, Netherlands)	N/A
MB-Ruler	Freeware https://www.markus-bader.de/MB-Ruler/download.php	N/A
InVivoStat	Freeware https://invivostat.co.uk/	N/A
Graphpad Prism	Graphpad software (CA, USA)	N/A
Cosinor	Freeware https://www.circadian.org/softwar.html	N/A

**Other**		

HCA Home Cage Analysis System	Actual Analytics (UK)	N/A
Phenomaster System	TSE Systems (Bad Homburg, Germany)	N/A
Lumicycle 32	Actimetrics (IL, USA)	N/A
Echo MRI	Echo Medical Systems (Houston, USA)	N/A

### Resource availability

#### Lead contact

Further information and requests for resources and reagents should be directed to and will be fulfilled by the Lead Contact, Patrick Nolan (p.nolan@har.mrc.ac.uk).

#### Materials availability

All unique reagents generated in this study are available from the Lead Contact with a completed Materials Transfer Agreement.

### Experimental model and subject details

#### Mice

All animal studies were performed under the guidance issued by the Medical Research Council in Responsibility in the Use of Animals for Medical Research (July 1993) and Home Office Project License 30/3206, with local ethical approval (MRC Harwell and MRC LMB AWERBs). When not being tested, mice were group-housed in individually ventilated cages under 12/12 h light/dark conditions with food and water available *ad libitum*. All animals were bred on a congenic C57BL/6J background. Floxed *Zfhx3* mice, *Zfhx3*^*Tm1.1Jtd*^, were kindly provided by Dr Jin-Tang Dong (Emory University) (Sun et al., 2012). The *Tg(Six3-cre)69Frty* line was created by Dr Yas Furuta (Furuta et al., 2000) and imported from Joseph Bedont (Johns Hopkins University). Only hemizygous Cre-driver animals were used for this study. For *ex vivo* slice studies, all combinations of genotypes were generated in crosses to PER2:LUC mice (*Per2*^*Tm1Jt*^) (Yoo et al., 2004). Mice were genotyped for *Zfhx3* using a qPCR copy count Taqman assay. WT primers: AAGAAGCGATAAGCTAACACCAGG, and ACGCCAAAGGTTGAGGAGAATG, probe sequence: TTAAAGGAATTCACGGGGTTAGGGC. Mutant primers: GCCATAACTTCGTATAATGTATGCTATACG and ACGCCAAAGGTTGAGGAGAATG, probe sequence: TTATAAGCTTACGGGGTTAGGGCTGT. For genotyping the Cre line, the same copy count assay was used with the following primers: CCATGGCTCCCAAGAAGAAGAG and CCTGGCGATCCCTGAACATG, probe sequence: TGTCCAATTTACTGACCGTACACCAA. PER2:LUC mice were genotyped as previously described (Yoo et al., 2004) using CTGTGTTTACTGCGAGAGT (FW) and GGGTCCATGTGATTAGAAAC (Rev) for the WT allele and TAAAACCGGGAGGTAGATGAGA as a reverse primer with WT (FW) for the *Luc* knockin allele (Table S1). Adult male and female mice were used as indicated, no sex differences affecting data analysis were identified and values were pooled for males and females.

### Method details

#### General circadian phenotyping

Wheel-running activity was monitored in singly housed animals (Banks and Nolan, 2011). Briefly, adult male and female mice (aged >6 weeks) were housed in cages containing running wheels, placed in light controlled chambers and wheel running activity monitored via ClockLab (Actimetrics). Animals were monitored for five days in a 12 hour light/12 hour dark cycle (100 lux light intensity) followed by two weeks in constant darkness. Video tracking analysis of masking behaviour was performed to complement wheel-running experiments (Banks et al., 2015). Briefly, adult male and female mice (aged >6 weeks) were singly housed and placed in light controlled chambers with Near-Infrared miniature CCD cameras positioned above the cages (Maplin, UK). Monitoring during dark periods was performed using infrared illumination. Mice were allowed to acclimatize to the home cage for 24 hours in a 12-hour light/dark cycle (100 lux light intensity) before data collection. Mice were subjected to a 1-hour light pulse at ZT14 and were video monitored throughout the 24hr light/dark cycle including the pulse. Video files were uploaded to ANYmaze video analysis software (Stoetling).

#### Feeding entrainment

Experiments were performed using 6 months old animals. The cohorts used were: 6 female and 14 male control animals (*Zfhx3*^Flox/+^; Six3-Cre^-^, *Zfhx3*^Flox/+^; Six3-Cre^+^ or *Zfhx3*^Flox/Flox^; Six3-Cre^-^ genotypes); 5 female and 2 male *Zfhx3*^Flox/Flox^; Six3-Cre^+^ animals. Mice were initially housed for five days in a 12 hour light/12 hour dark cycle (lights on at 0700) followed by 5 days in constant darkness with access to food *ad libitum*. Subsequently, mice underwent a restricted feeding schedule. Mice had access to food for 12 hours (8am to 8pm) for 3 days but this was reduced to 8 hours (8am to 4pm) for the following 3 days, and then to 6 hours (8am to 2pm) for the remainder of the screen. For the final week of the screen, mice were then placed on *ad libitum* feeding while being maintained in constant darkness. Throughout, animals were monitored for wheel-running activity as described above. Anticipatory activity was calculated by recording average total activity counts for 3 hours prior to food availability and also averaged for the total time under 6 hours restricted feeding. The corresponding 3 hours interval in the days prior to food restriction, when mice were on *ad libitum* food, was taken and averaged for comparison.

#### Home cage activity monitoring

Female mice (3 months of age) were housed in trios composed of two control genotypes (*Zfhx3*^Flox/+^; Six3-Cre^-^ or *Zfhx3*^+/+^; Six3-Cre^+^) and one *Zfhx3*^Flox/Flox^; Six3-Cre^+^ animal. Group-housed mice were tagged with RFID microchips inserted into the abdominal cavity and left to recover for three days in the home cage. Chipped mice were then placed in the Home Cage Analysis system (HCA, Actual Analytics, Edinburgh). Video data were captured while location tracking of RFID co-ordinates was performed using a gridded array of RFID sensors beneath the cage base (Bains et al., 2016). The activity of individual mice was recorded continuously for 72 hours.

#### Open field activity

Adult male mice were placed into one corner of a walled arena (45 cm × 45 cm) and allowed to explore for 30 min. Animal movements and position were tracked using EthoVision XT analysis software (Noldus).

#### Nissl and *in situ* hybridisation staining

Nissl staining and *in situ* hybridisation were performed on frozen coronal brain sections from adult males cut at 14μm and mounted of positively-charged slides (Oliver et al., 2012). For Nissl staining, slides were incubated in 4% PFA for 10 min then washed with PBS. Subsequently, sections were stained in 0.1% Cresyl Violet solution for 15 min. Sections were then rinsed with dH_2_0 and washed in 70% ethanol before being dehydrated in 100% ethanol. Slides were then cleared in Xylene and mounted. For *in situ* hybridization, DIG-labelled RNA probes were synthesised from regions of the genes *Avp* (1–490 bp of accession number NM_009732.1) and *Vip* (191–715 bp of NM_011702.1) first cloned into pCR4-TOPO (SigmaAldrich). For sample preparation, slides were incubated in 4% PFA in PBS at RT for 15 min and washed in PBS. Slides were then placed into acetylation mix (450 ml DEPC H2O, 5.7 ml triethanolamine, 780 μl concentrated hydrochloric acid) while 1 ml of acetic anhydride was added dropwise over 10 min. After PBS washing, 500 μl hybridisation solution (2.5 ml Deionisied Formamide 100%, 100 μl *E. Coli* tRNA (10 mg/ml) (Merck, UK), 100 μl 1X Denhardt's 50 X (Merck, UK), 0.5 g Dextran sulphate, 0.175 g NaCl, 0.012 g SDS, 10 μl 0.5M EDTA pH 8.0, DEPC H_2_O up to 5ml) was added to each slide and left to incubate for 1 hour at RT. This was replaced with 200 μl hybridisation mix containing 100 ng of probe. Parafilm-covered samples were incubated for 16-20 hours at 60°C in a chamber containing 5 X SSC, 50% Formamide solution to maintain humidity. Slides were then washed at 70°C in 5 X SSC for 5 min, then 0.2 X SSC at 70°C for 1 hour followed by 5 minutes in buffer B1 (0.15M NaCl, 0.1M Tris-HCl pH 7.6) at RT. For labelling, slides were incubated in blocking solution (1% Blocking Reagent (Roche) in buffer B1) for 1 hour at RT and then with blocking solution containing anti-DIG antibody (ThermoFisher Scientific, 1:5000) overnight at 4°C. Slides were then washed in buffer B1 and then in buffer B2 (0.1M NaCl, 0.1M Tris-HCl pH 9.5, 0.05M MgCl_2_) each for 5 min at RT. The signal was detected using 2% NBT/BCIP reagent (Roche) in buffer B2. The slides were developed for 6 hours (*Avp*) or 16 hours (*Vip*). Slides were mounted in 90% glycerol for imaging.

#### Immunofluorescence

Brains from adult males were dissected, immediately embedded in OCT compound (VWR international, USA) and snap frozen. 12 μM sections were then fixed in 4% paraformaldehyde, washed in PBS and then blocked in 5% normal goat serum made up in 0.5% PBS-Triton. Anti-ZFHX3 raised in Rabbit (Parsons et al., 2015) was diluted 1:1000 in the blocking solution and primary incubation was 48 hours at 4°C. Following washing in PBS, Anti-Rabbit 488 raised in goat (Abcam, UK) was diluted 1:200 in PBS and incubated for 2 hours at room temperature. Following more washes in PBS, slides were coverslipped using ProLong Gold Antifade mountant with DAPI (Life Technologies, UK). Sections were imaged using an inverted confocal microscope.

#### Intraocular injection

*Zfhx3*^+/+^; Six3-Cre^-^ control (n = 3, female), *Zfhx3*^Flox/Flox^; Six3-Cre^+^ (n = 3, 2 female and 1 male) and *Zfhx3*^Flox/+^; Six3-Cre^+^ (n = 3, 2 male and 1 female) mice (aged 4-6 months) were anaesthetised via intraperitoneal (i.p.) injection of Dormitor (1 mg/kg) and Ketamine (60 mg/kg) with administration of i.p. Antisedan (5 mg/kg) to reverse anaesthesia once surgery was completed (Wong et al., 2018). To identify retinofugal projections, mice received intraocular injections (2 μl) of fluorescently tagged Cholera Toxin subunit B conjugated with Alexa Fluor 488 (6.25 μg/ml; Cat. No. C34775; Thermo Fisher Scientific) and 594 (5 μg/μl; Cat. No. C34777), injected into the left and right eyes respectively, following administration of 1% tropicamide followed by 2.5% phenylephrine hydrochloride drops to dilate the pupils. The 30g hypodermic needle was held in place for 5 s after delivery of the tracers. Immediately following surgery Visotears and the local anaesthetic, proxymetacaine, were applied to the eyes, then daily chloramphenicol eye drops to prevent infection. After 72 hours, mice were culled and brains dissected, fixed in 4% paraformaldehyde (PFA) in phosphate buffer for 8 hours at 4°C, cryopreserved overnight in 20% sucrose in PBS and then sectioned (40 μm) on a freezing sledge microtome (Bright Instruments, UK). Sections were mounted onto slides in VectaShield with DAPI (Vector Labs) and confocal microscopy (Zeiss 780 inverted confocal system) was used to visualise the fluorescence signal in the retinal ganglion cell axonal projections to the SCN and lateral geniculate nucleus (LGN).

#### Pupillometry

Measurements of pupillary light responses were recorded using a custom-built system (Banks et al., 2015). Mice (males, 4 to 5 months of age, n = 8 *Zfhx3*^Flox/Flox^; Six3-Cre^+^, n = 15 controls (genotypes: *Zfhx3*^Flox/+^; Six3-Cre^-^ and *Zfhx3*^Flox/+^; Six3-Cre^-^)) were dark adapted for 1–2 hours prior to testing. Images of the consensual pupillary light response were measured in response to an irradiant light stimulus. After brief baseline measurements of the dark-adapted pupil (2 s), the left eye was exposed to the light stimulus for 10 s. Each animal was tested on multiple occasions to minimise any artefacts due to handling. Images were analysed using MB-Ruler to measure pupil diameter and pupil area calculated (Markus Bader, Germany).

#### Visual acuity

Visual acuity was assessed by head tracking response to a virtual reality optokinetic system (Banks et al., 2015). Mice (n = 2 female and n = 1 male *Zfhx3*^Flox/Flox^; Six3-Cre^+^ animals and n = 5 female and n = 4 male controls (genotypes: *Zfhx3*^Flox/+^; Six3-Cre^+^, *Zfhx3*^+/+^; Six3-Cre^+^, *Zfhx3*^Flox/+^; Six3-Cre^-^ and *Zfhx3+*^+/+^; Six3-Cre^-^)) were acclimatised for 30 min to the phenotyping room prior to starting the test. Mice were then individually placed onto a raised circular platform of 8 cm diameter in the centre of four screens each displaying the same black and white vertical bar pattern. The mouse was allowed to settle for 30 s before starting the test. The frequencies of the stripes used were 0.25 cycles/degree (subtending an angle of 2° when viewed from the centre of the drum), 0.125 cycles/degree (4°), or 0.0625 cycles/degree (8°). The pattern was rotated anticlockwise for 30 s at a rotation speed of two revolutions per minute (12°/sec) to assess right eye ability and repeated clockwise to assess left eye ability. During the rotations, the mouse was observed for its head tracking response. The test started with the 2° stripe and if no head tracking response was observed, the stripe was increased to 4° and then 8°.

#### qRT-PCR

Adult mutants and littermate controls (ZT2, n = 5, 2 males and 3 females per genotype; ZT14, n = 5, 3 males and 2 females per genotype) on a 12 hour light/12 hour dark cycle were sacrificed at one of two time points (ZT2 or ZT14) and livers harvested, dissected, immediately snap frozen on dry ice and stored at −80°C. RNA was extracted using the Maxwell 16 Instrument (Promega, UK) and the Maxwell LEV simplyRNA tissue kit (Promega, UK) as per instructions. RNA was resuspended in 40 μl water. cDNA synthesis was performed using the High Capacity cDNA RT kit (Thermo Fisher Scientific) starting with 2 μg of total RNA. cDNA for qRT-PCR amplification was used at a final concentration of 10 ng per well. Technical replicates were run for all samples. Fast Sybr Green mastermix (Thermo Fisher Scientific) was used in a final volume of 20 μl. Primers were used at a final concentration of 360 nM (Table S1). Fold changes were calculated using the ΔΔC_t_ method using the 7500 Software v2.0.6 and normalised using actin as a housekeeping control.

#### Metabolic phenotyping

Prior to being housed in metabolic caging, body composition analysis was performed on mice aged 3-4 months (males, n = 10 *Zfhx3*^Flox/Flox^, Six3-Cre^-^; n = 6 *Zfhx3*^Flox/Flox^, Six3-Cre^+^) using an Echo MRI whole body composition analyser (Echo Medical System, USA). Percentage fat and lean mass were recorded as well as weight measured. Mice (males, 3-4 months old, n = 11 *Zfhx3*^Flox/Flox^, Six3-Cre^-^; n = 8 *Zfhx3*^Flox/Flox^, Six3-Cre^+^) were then individually housed in PhenoMaster cages (TSE Systems, Bad Homburg, Germany) for 3 days. To mitigate for any stress effects of being introduced into a novel environment, analysis of energy intake/expenditure related data was carried out using the final 24 hours of this time. The cage system includes a photobeam-based activity monitoring system that records ambulatory movements in the horizontal and vertical planes. Food and water were available *ad libitum* throughout testing. Measures for activity/distance travelled, O_2_ consumption, CO_2_ production, respiratory exchange ratio (RER) and energy expenditure (EE) were determined.

#### Luciferase recordings

The *Per2::Luc* reporter was crossed to experimental lines to generate all genotypes expressing the reporter construct. Luminescence recordings were performed on tissue samples from adult male mutants and controls (Yamazaki and Takahashi, 2005). Briefly, experimental animals were sacrificed at 3-4 months of age and the brain and liver were dissected into ice cold dissection buffer (HBSS, supplemented with Penicillin–streptomycin and HEPES). Small pieces of the liver were hand-dissected and placed onto culture inserts and then into 35mm culture dishes. Samples were covered in recording media (DMEM, supplemented with sodium bicarbonate, HEPES, Penicillin–streptomycin, B27 and Beetle luciferin potassium salt) and the culture dish sealed using a glass coverslip. Samples were placed into a LumiCycle luminometry recorder (ActiMetrics) and recordings taken for at least four days.

### Quantification and statistical analysis

Statistical analyses were performed using In-Vivo Stat (v3.7) and GraphPad Prism Software (v7). Shapiro-Wilk testing was used to test for normality of data to decide whether parametric or non-parametric analysis was to be conducted. If data were deemed normally distributed then regular ANOVA or student's t-test were used where applicable (with the exception of daily average O_2_ consumption, CO_2_ production and energy expenditure, in which an ANCOVA was performed with lean mass as a covariant). If data were deemed not to be normally distributed then Kruskal-Wallis ANOVA or non-parametric t test was used where applicable. Following ANOVA, Tukey and Dunn's multiple comparison testing was performed for parametric and non-parametric data respectively. Rhythmicity analysis was performed using the Cosinor program available at https://www.circadian.org/softwar.html. A dataset was deemed to be arrhythmic if no significant rhythm was detected using this software (p > 0.05).

## Data Availability

• This paper does not report original code. • Source data related to this paper is available upon request. • Any additional information required to reanalyze the data reported in this paper is available from the lead contact upon request.

## Supplemental information

Documents S1. Figures S1–S7 and Table S1
