# Supplementary material for: Zfhx3-mediated genetic ablation of the SCN abolishes light entrainable circadian activity while sparing food anticipatory activity
Source: iScience. 2021 Sep 16;24(10):103142. doi: 10.1016/j.isci.2021.103142 (PMC8487057; doi:10.1016/j.isci.2021.103142)
Supplement: Documents S1. Figures S1–S7 and Table S1 [file mmc1.pdf]

iScience, Volume *24*

Supplemental information

Zfhx3-mediated genetic ablation of the SCN abolishes light entrainable circadian activity while sparing food anticipatory activity

Ashleigh G. Wilcox, R. Sonia Bains, Debbie Williams, Elizabeth Joynson, Lucie Vizor, Peter L. Oliver, Elizabeth S. Maywood, Michael H. Hastings, Gareth Banks, and Patrick M. Nolan

A

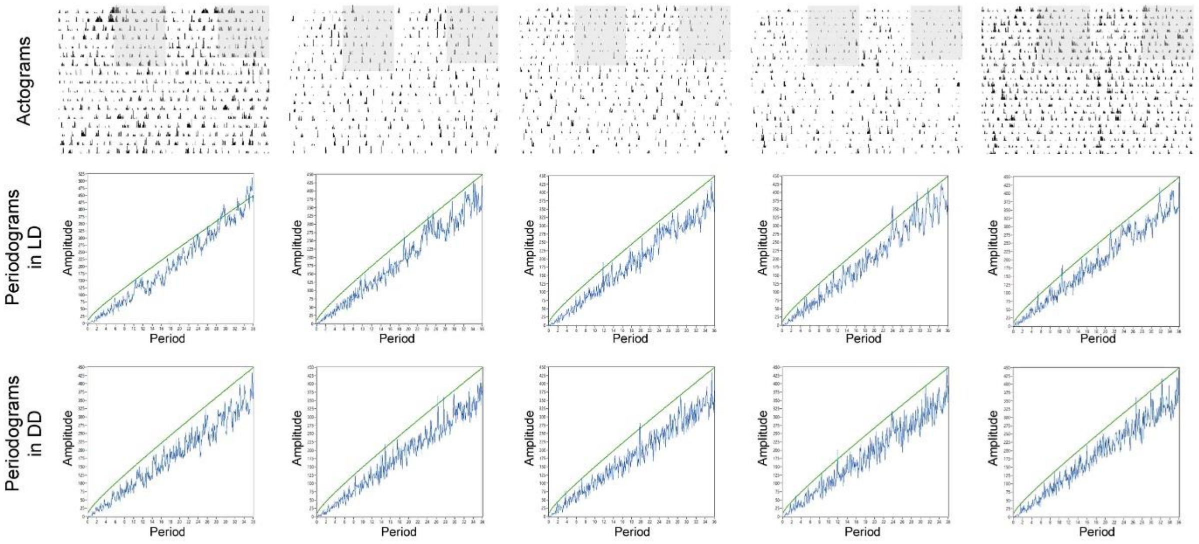

B

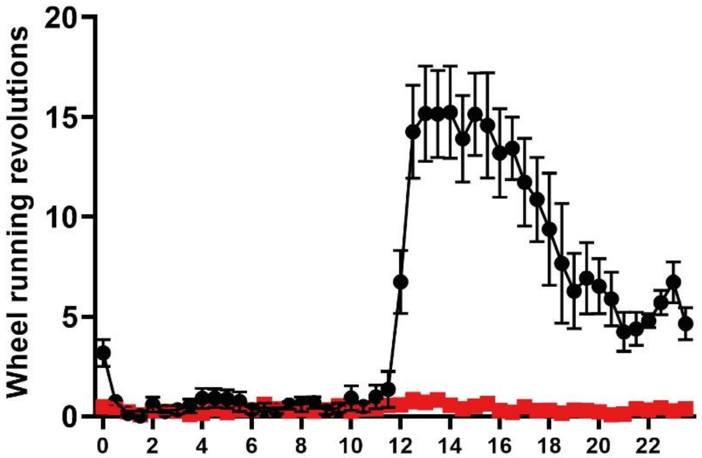

Figure S1. Arrhythmicity and wheel-running deficits in *Zfhx3*$^{\text{Flox/Flox}}$; *Six3*-Cre^{+} mutants, Related to Figure 1. (A) Additional double-plotted actograms and periodograms for *Zfhx3*$^{\text{Flox/Flox}}$; *Six3*-Cre^{+} mutants showing arrhythmicity in all conditions. **(B)** Line plots showing average (± SEM) wheel revolutions per time bin in *Zfhx3*$^{\text{Flox/Flox}}$; *Six3*-Cre^{-} control (black) and *Zfhx3*$^{\text{Flox/Flox}}$; *Six3*-Cre^{+} (red) mutant animals (n=10). Note that differences in wheel-running are confined to the dark phase in *Zfhx3*$^{\text{Flox/Flox}}$; *Six3*-Cre^{+} mutants.

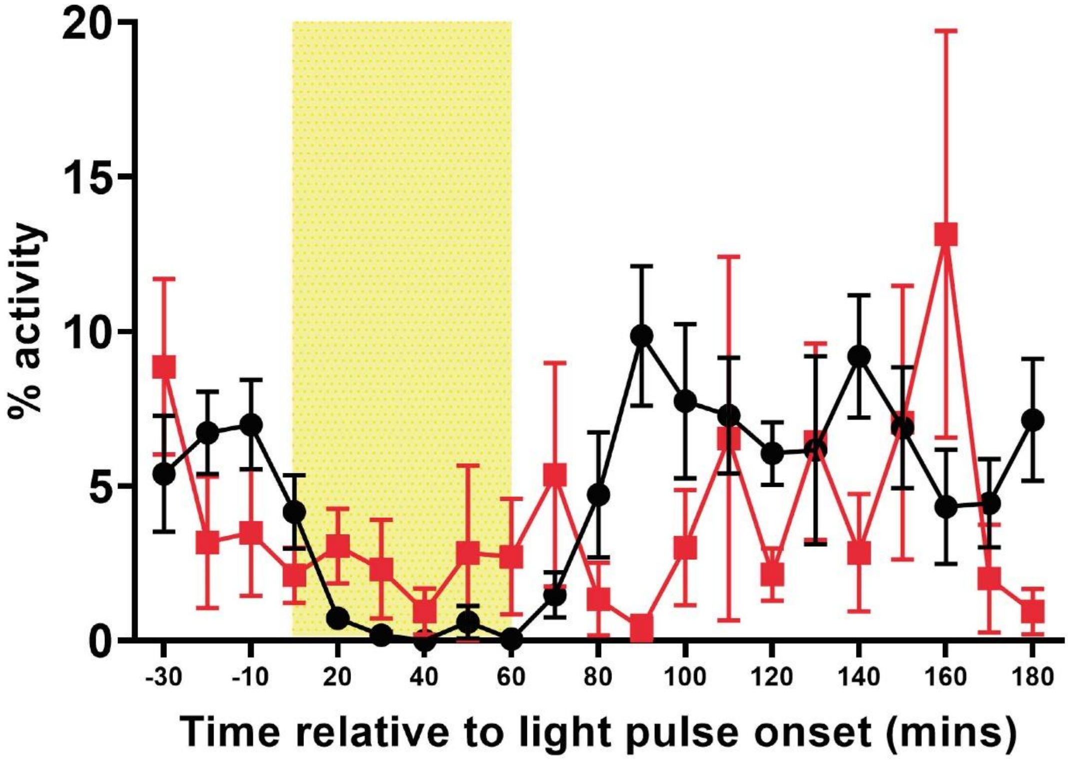

Figure S2. Light-induced suppression of activity is absent in *Zfhx3*$^{Flox/Flox}$; *Six3*-Cre^{+} mutants, Related to Figure 1. Line plot showing percentage activities in 10 minute bins preceding, during and following a 60-minute light pulse (yellow background) administered at ZT14 in *Zfhx3*$^{Flox/Flox}$; *Six3*-Cre^{-} control (black, n=10) and *Zfhx3*$^{Flox/Flox}$; *Six3*-Cre^{+} (red, n=6) mutant animals. Data are presented as mean ($\pm$ SEM). Repeated measures ANOVA showed no statistical differences.

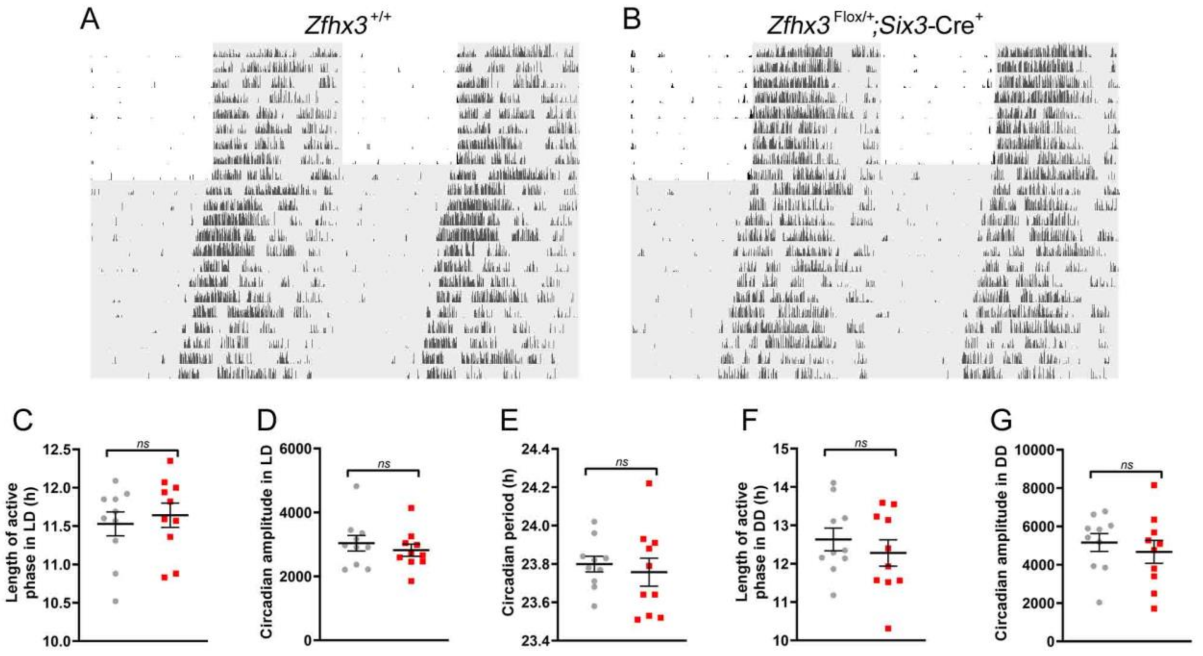

Figure S3. Heterozygous null animals show no significant circadian phenotypes compared to controls, Related to Figure 1. Representative double-plotted actograms for *Zfhx3*$^{Flox/Flox}$; *Six3*-Cre^{-} control (**A**) and *Zfhx3*$^{Flox/+}$; *Six3*-Cre^{+} mutant animals (**B**). Animals were initially housed in 12:12 light-dark (LD) cycles for eight days and then subjected to two weeks of constant darkness (DD). Shaded parts of actograms represent lights-off, wheel-running is represented as vertical black bars. Analysis of this data by ANOVA demonstrated no significant differences (ns) between controls and *Zfhx3*$^{Flox/+}$; *Six3*-Cre^{+} mutants in circadian parameters including, the length of the active phase in the LD cycle (**C**), circadian amplitude in LD cycles (**D**), circadian period (**E**), length of the active phase in DD (**F**) and circadian amplitude in DD (**G**). Data are presented as mean (± SEM) and individual values are shown.

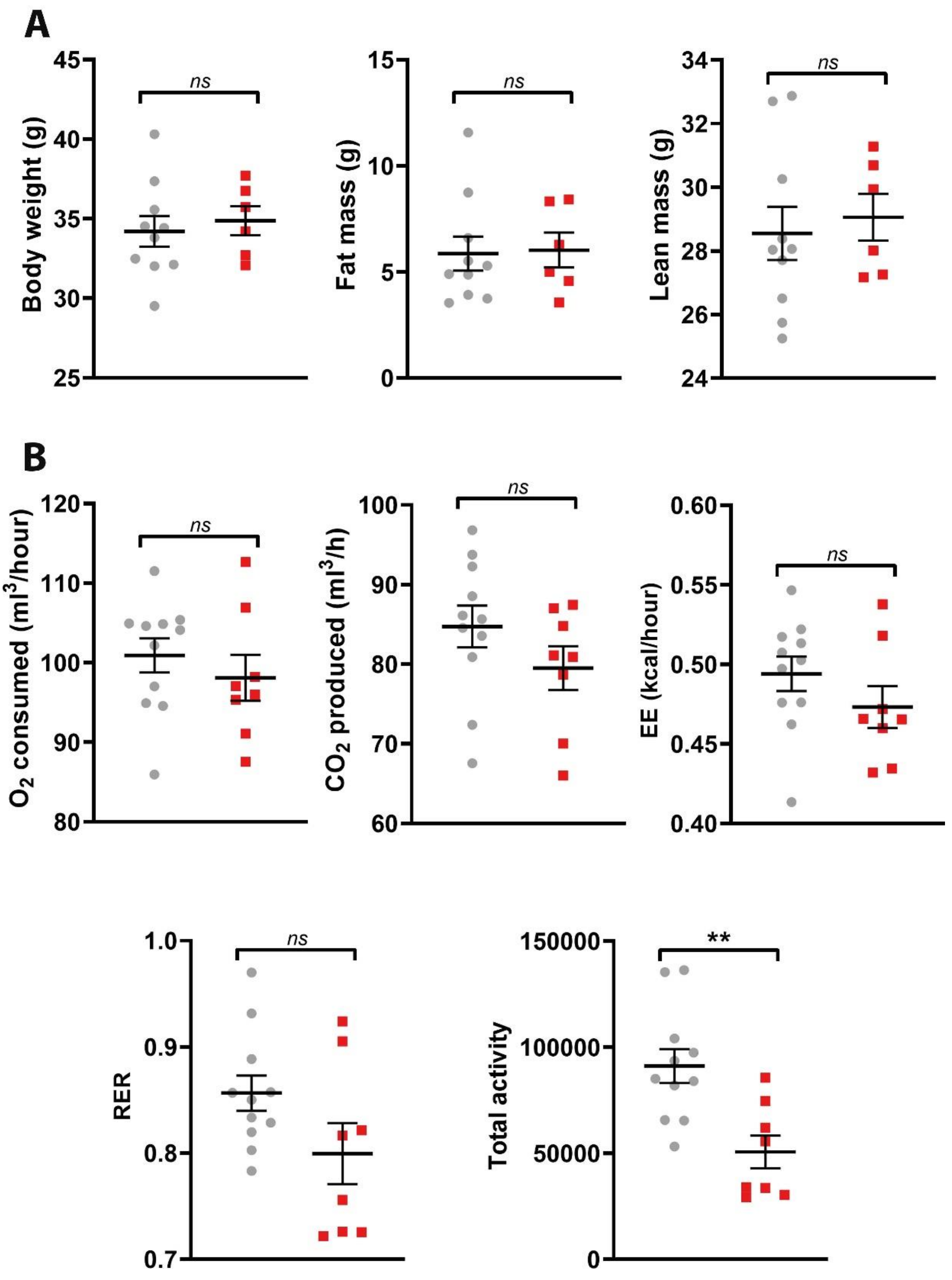

Figure S4. Body composition analysis and daily metabolic scores in controls and *Zfhx3*$^{Flox/Flox}$; *Six3*-Cre^{+} mutants, Related to Figure 3. Body weight, fat mass and lean mass in *Zfhx3*$^{Flox/Flox}$; *Six3*-Cre^{-} control (black, n=10) and *Zfhx3*$^{Flox/Flox}$; *Six3*-Cre^{+} (red, n=6) mutant animals (**A**). Daily O_2 consumption, CO_2 production, EE, RER and total activity in *Zfhx3*$^{Flox/Flox}$; *Six3*-Cre^{-} control (black, n=11) and *Zfhx3*$^{Flox/Flox}$; *Six3*-Cre^{+} (red, n=8) mutant animals (**B**). Data are presented as mean ($\pm$ SEM) and individual values are shown. Data analysed using ANOVA and ANCOVA, *p<0.01.

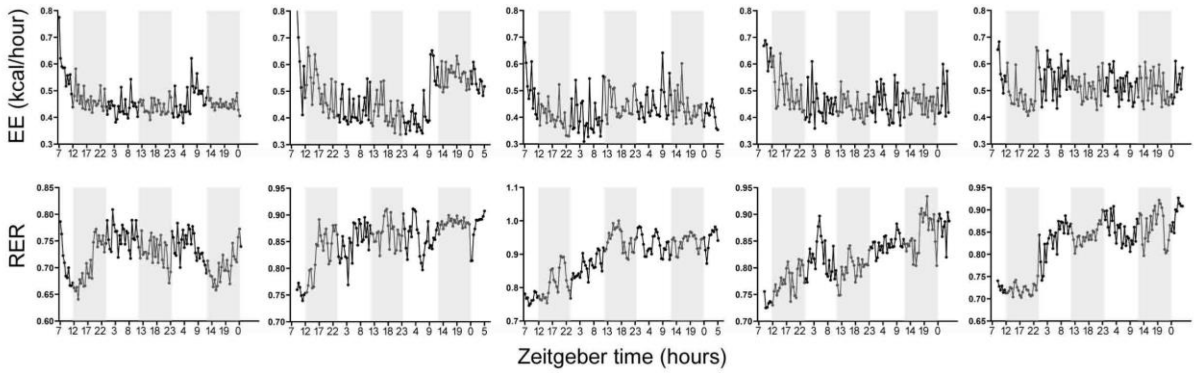

Figure S5. Metabolic function in individual *Zfhx3*$^{Flox/Flox}$; *Six3*-Cre^{+} animals, Related to Figure 3. Individual plots of hourly measures for EE and RER in *Zfhx3*$^{Flox/Flox}$; *Six3*-Cre^{+} mutants over the three day course of metabolic recording.

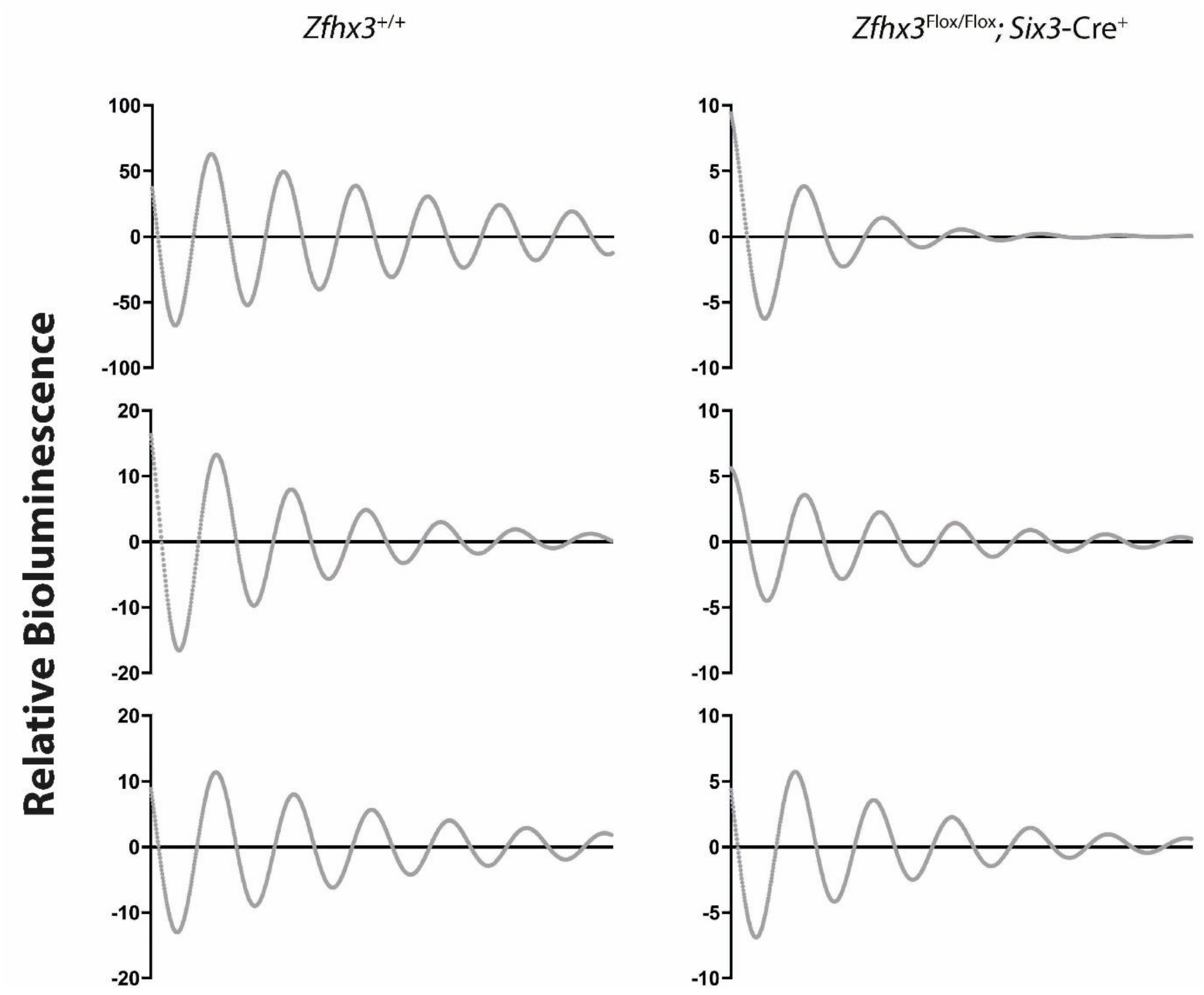

Figure S6. Individual Per2::Luc bioluminescence traces of liver slices from *Zfhx3*$^{\mathbf{Flox/Flox}}$; *Six3*-Cre^{+} and control animals, Related to Figure 3.

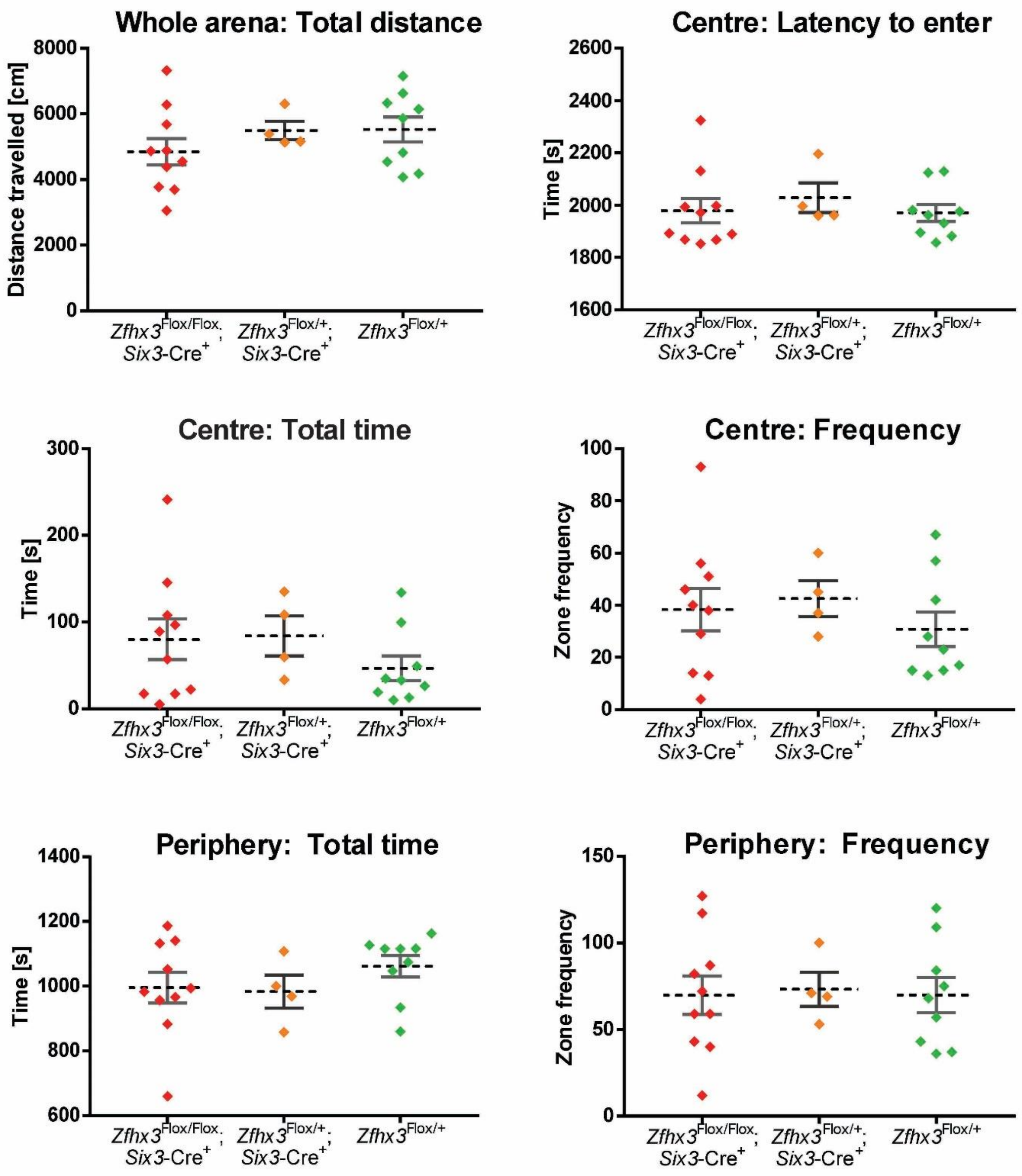

Figure S7. Open field activity in *Zfhx3*$^{\text{Flox/Flox}}$; *Six3*-Cre^{+} animals, Related to Figure 5. Open field parameters measured from *Zfhx3*$^{Flox/Flox}$*; Six3-Cre*$^{+}$ (red, n=10), *Zfhx3*$^{Flox/+}$*; Six3-Cre*$^{+}$ (orange, n=4) and *Zfhx3*$^{Flox/+}$ (green, n=9) mice. No statistical differences between groups were found in total distance travelled, latency to centre, total time in the centre, frequency of entries to the centre, total time in arena periphery and frequency of periphery entries. Dashed lines denote mean of group data, error bars indicate SEM of group data.

Supplementary Table 1. PCR Primer Sequences, Related to STAR Methods

Primer Name	Sequence 5’ to 3’
**Zfhx3 Taqman wildtype FW**	AAGAAGCGATAAGCTAACACCAGG
**Zfhx3 Taqman wildtype Rev**	ACGCCAAAGGTTGAGGAGAATG
**Zfhx3 Taqman wildtype probe**	TTAAAGGAATTCACGGGGTTAGGGC
**Zfhx3 Taqman mutant FW**	GCCATAACTTCGTATAATGTATGCT ATACG
**Zfhx3 Taqman mutant Rev**	ACGCCAAAGGTTGAGGAGAATG
**Zfhx3 Taqman mutant probe**	ACGCCAAAGGTTGAGGAGAATG
**Cre Taqman FW**	CCATGGCTCCCAAGAAGAAGAG
**Cre Taqman Rev**	CCTGGCGATCCCTGAACATG
**Cre Taqman probe**	TGTCCAATTTACTGACCGTACACCA A
**Per2Luc Fw**	CTGTGTTTACTGCGAGAGT
**Per2Luc Rev**	GGGTCCATGTGATTAGAAAC
**Per2Luc knockin Rev**	TAAAACCGGGAGGTAGATGAGA
**Actin FW qPCR**	CGATGCCCTGAGGCTCTTT
**Actin Rev qPCR**	TGGATGCCACAGGATTCCAT
**Arntl FW qPCR**	CCGTGCTAAGGATGGCTGTT
**Arntl Rev qPCR**	TTGGCTTGTAGTTTGCTTCTG
**Cry1 FW qPCR**	GCTATGCTCCTGGAGAGAACGT
**Cry1 Rev qPCR**	TGTCCCCGTGAGCATAGTGTAA
**Dbp FW qPCR**	GAGCCTTCTGCAGGGAAACA
**Dbp Rev qPCR**	GCCTTGCGCTCCTTTTCC
**Per2 FW qPCR**	AGCTACACCACCCCTTACAAGCT
**Per2 Rev qPCR**	GACACGGCAGAAAAAAGATTTCTC
